# Molecular docking, synthesis and biological significance of pyrimidine analogues as prospective antimicrobial and antiproliferative agents

**DOI:** 10.1186/s13065-019-0601-z

**Published:** 2019-07-09

**Authors:** Sanjiv Kumar, Archana Kaushik, Balasubramanian Narasimhan, Syed Adnan Ali Shah, Siong Meng Lim, Kalavathy Ramasamy, Vasudevan Mani

**Affiliations:** 1 Faculty of Pharmaceutical Sciences, Maharshi Dayanand University, Rohtak, 124001 India; 2 Faculty of Pharmacy, Universiti Teknologi MARA (UiTM), 42300 Bandar Puncak Alam, Selangor Darul Ehsan Malaysia; 3 Atta-ur-Rahman Institute for Natural Products Discovery (AuRIns), Universiti Teknologi MARA, 42300 Bandar Puncak Alam, Selangor Darul Ehsan Malaysia; 4 Collaborative Drug Discovery Research (CDDR) Group, Pharmaceutical Life Sciences Community of Research, Universiti Teknologi MARA (UiTM), 40450 Shah Alam, Selangor Darul Ehsan Malaysia; 5 Department of Pharmacology and Toxicology, College of Pharmacy, Qassim University, Buraidah, 51452 Kingdom of Saudi Arabia

**Keywords:** Pyrimidine analogues, Antibacterial activity, Anticancer activity, Docking study

## Abstract

Pyrimidine nucleus is a significant pharmacophore that exhibited excellent pharmacological activities. A series of pyrimidine scaffolds was synthesized and its chemical structures were confirmed by physicochemical and spectral analysis. The synthesized compounds were evaluated for their antimicrobial potential towards Gram positive and negative bacteria as well as fungal species. They were also assessed for their anticancer activity toward a human colorectal carcinoma cell line (HCT116). Whilst results of antimicrobial potential revealed that compounds **Ax2**, **Ax3**, **Ax8** and **Ax14** exhibited better activity against tested microorganisms, the results of antiproliferative activity indicated that compounds **Ax7** and **Ax10** showed excellent activity against HCT116. Further, the molecular docking of pyrimidine derivatives **Ax1**, **Ax9** and **Ax10** with CDK8 (PDB id: 5FGK) protein indicated that moderate to better docking results within the binding pocket. Compounds **Ax8** and **Ax10** having significant antimicrobial and anticancer activities may be selected as lead compounds for the development of novel antimicrobial and anticancer agent, respectively.
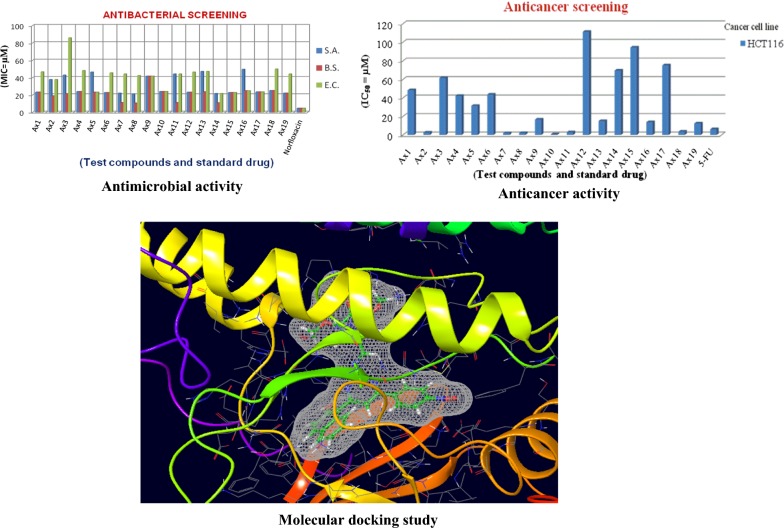

## Introduction

Drug designing is a technique of searching and developing new molecules that exert specific action on a human kind [1]. The figure of multidrug resistant microbial infections is growing day by day which indicated that it is crucial to develop new class of antimicrobial drugs [2]. Tumor is a severe health issue and 2nd leading/most reason for mortality in the globe. It is caused by deregulation of the cell cycle which results in failure of cellular differentiation and unrestrained cellular growth [3, 4]. So, it is necessary to develop and synthesize new bioactive molecules whose chemical structure and mode of action are noticeably differing from the available agents [5].

Discovery of drug is a slow, lengthy costly and interdisciplinary procedure but the new developments have transformed the methods by which researchers generate new drug molecules e.g. CADD tool overcomes the cost of drug design up to 50% [1]. Molecular docking technique is used to understand the (i) drug-receptor interaction (ii) binding affinity (iii) orientation and approach of drug molecules to the target site. The main objectives of docking study are precise structural modeling, correct prediction of activity. It presents the most promising vision of drug–receptor interaction and generates a new rational approach to drug design [6]. RMSD is the average distance between the atoms of superimposed structures. This value is widely used parameter to rank the performance of docking methods. If the docked ligand shows < 2.0 Å RMSD value with the crystallographic ligand, it is considered as a successful docking. To calculate the relative free energy, an accurate MM-GBSA binding affinity computation can also be applied [7, 8].

Cyclin-dependent kinases play a significant role in the control of cell cycle. These holoenzymes have both catalytic (CDK) and regulatory (cyclin) subunits but present as higher order complexes that include additional proteins and are arbitrated by two classes of enzymes i.e. cyclin D- and E. The D-type cyclins (D1, D2 and D3) bind with two different catalytic sites (CDK4 and CDK6) to yield six possible holoenzymes that articulated in tissue-specific models [9].

CDKs are a class of enzymes that controls the cell cycle and are novel targets for prospective anticancer drugs [10]. A series of pyrimidines bearing 2-arylamino substituents was developed and screened for CDK1 and CDK2 inhibitory effect by Sayle et al. [11]. The SAR of 4-cyclohexylmethoxy-pyrimidines (inhibitors of CDK2) was explored [12]. The progression, transcription and other related functions of cell cycle are regulated by CDK8 that is a heterodimeric kinase protein. The carboxyterminal domain of RNA polymerase II is also phosphorylated by CDK-8. Hence, the inhibition of CDK-8 protein may be essential for regulating tumor [6, 13].

Pyrimidine is a heterocyclic nucleus containing nitrogen atom at 1 and 3 positions. It is the structural unit of DNA and RNA is an important molecule also plays a very significant role in the field of medicinal chemistry [14]. Pyrimidine is reported to have antimicrobial [15], anticancer [16, 17], anti-inflammatory [18], antioxidant [19], analgesic [20] and antiviral [21] and antimalarial [22] potentials. Number of marketed drugs contains pyrimidine ring such as proquazone (anti-inflammatory); idoxuridine (antiviral); trimethoprim (antibacterial); zidovudine (anti-HIV); pyrimethamine (antimalarial) and capecitabine (antiproliferative).

In the present study we have planned to synthesize heterocyclic pyrimidine analogues and evaluate their antimicrobial, antiproliferative and docking study.

## Results and discussion

### Chemistry

**Scheme 1:**
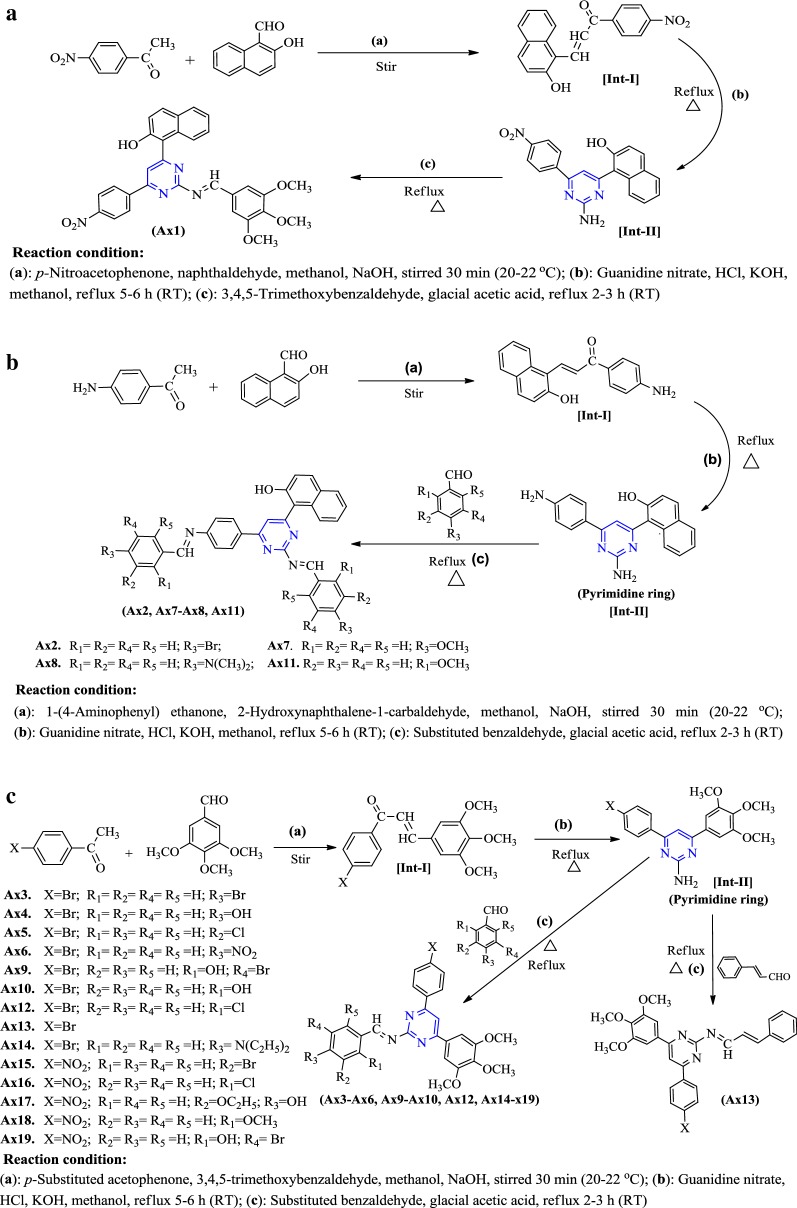
Synthesis of heterocyclic pyrimidine derivatives (**Ax1–Ax19**)

Synthesis of heterocyclic pyrimidine analogues followed the general procedure discussed in synthetic Scheme 1. The reaction of *p*-substituted acetophenone with substituted benzaldehyde resulted in the formation of Int- I. The resulted compound was treated with guanidine nitrate to yield pyrimidine ring (Int-II), which on reaction with corresponding substituted benzaldehyde in presence of glacial acetic acid yielded the final derivatives (**Ax1**–**Ax19**). The molecular scaffolds of the developed pyrimidine derivatives (**Ax1**–**Ax19**) were established by physicochemical properties (Table 1) and NMR, FTIR, MS spectra and elemental analysis (Table 2). The IR spectrum of synthesized compound showed bands around 2934–3093 cm^−1^ and 1462–1595 cm^−1^ which indicate the C–H and C=C group in aromatic nucleus, respectively. The Ar–Cl group in compounds **Ax5**, **Ax12**, **Ax16** were displayed stretches in the scale of 712–757 cm^−1^. The IR str. vibrations at 512–628 cm^−1^ in the spectral data of compounds displayed the Ar–Br group at *p*-position of the aromatic nucleus. The existence of Ar-OCH_3_ in synthesized analogues is established by absorption band around 1177–1276 cm^−1^. The appearance of IR str. 1550–1685 cm^−1^ in the compounds (**Ax1**–**Ax19**) specified the existence of N=CH group. The Ar-NO_2_ group in compounds **Ax1**, **Ax6** and **Ax15**–**Ax19** were displayed by symmetric Ar-NO_2_ str. in the scale of 1345–1462 cm^−1^. The IR stretching 1270–1363 cm^−1^ of synthesized compounds specified the existence of C–N group. The impression of IR absorption band at 3231–3491 cm^−1^ in the spectral data of the molecules displayed the presence of Ar-OH group on the aromatic nucleus. The signals between 6.39 and 8.38 δ in NMR spectra are indicative of aromatic proton. The prepared derivatives exhibited singlet at 7.46–8.39 δ due to the presence of N=CH group in pyrimidine nucleus. Molecules displayed singlet at 7.56–7.91 δ due to the presence of –CH group in pyrimidine nucleus. The singlet at 3.71–3.87 δ indicated the presence Ar-OCH_3_. Compound **Ax8** exhibited singlet at 2.67 δ due to presence of –N(CH_3_)_2_ at the *p*-position. The compound **Ax14** exhibited quadrate at 3.38 δ and triplet at 1.14 δ due to presence of –N(C_2_H_5_)_2_ group at *p*-position. The ^13^C-NMR spectra of aromatic ring exhibited in the range of 102.0, 112.3, 117.3, 123.6, 124.4, 126.6, 126.3, 128.1, 129.3, 130.2, 133.2, 147.5, 153.2; pyrimidine nucleus exhibited around 111.5, 164.3, 168.2; N=CH group exhibited around 161.0; OCH_3_ group showed around 54.1, 60.8, 56.1. The elemental analysis (CHN) was found within ± 0.4% of the theoretical results of derivatives.

**Table 1:**
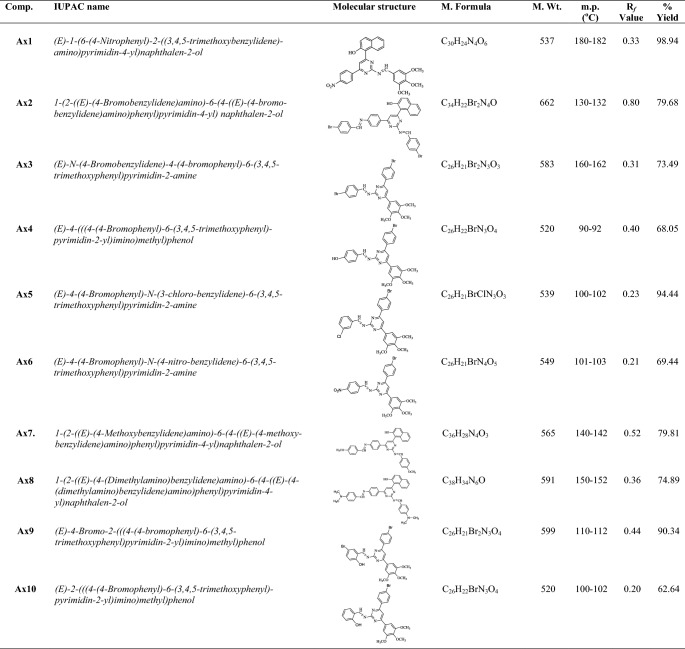

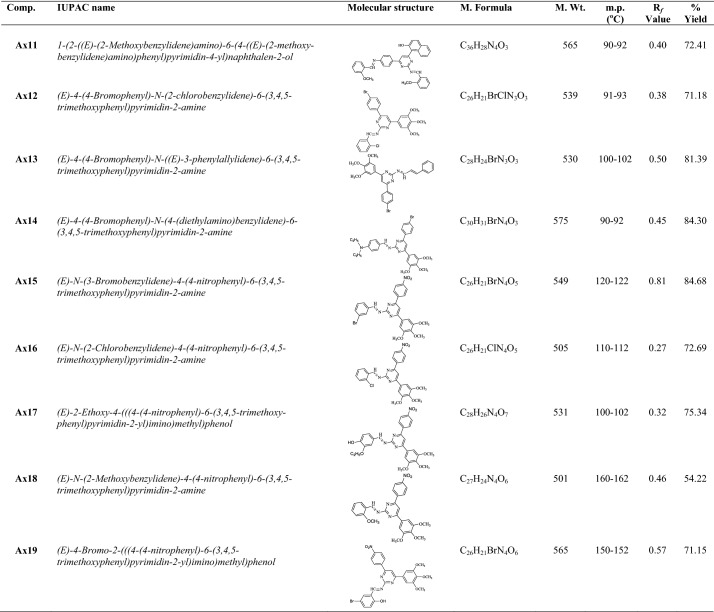
The physicochemical properties of synthesized pyrimidine derivatives

**Table 2:** Spectral data of synthesized pyrimidine compounds

Comp.	FT-IR (KBr cm^−1^)						C, H, N Analysis Calculated (Found); *m/z*—[M^+^ +1]	^1^H NMR (δ, DMSO)	^13^C NMR (δ, DMSO)
	C–H str.	C=C str.	N=CH str.	C–N str.	C–O–C str.	Other str.			
**Ax1**	3073	1592	1683	1277	1233	3369 (C–OH str.), 1347 (NO_2_ str.), 852 (C–N str., NO_2_)	Anal calc: C, 67.16; H, 4.51; N, 10.44; Found: C, 67.20; H, 4.55; N, 10.49; *m/z*: 538	6.98-8.19 (m, 12H, Ar–H), 3.77 (s, 9H, OCH_3_), 8.18 (s, 1H, N=CH), 7.70 (s, 1H, pyrimidine)	Aromatic nucleus (102.0, 112.3, 117.3, 123.6, 124.4, 126.6, 126.3, 128.1, 129.3, 130.2, 133.2, 147.5, 153.2), pyrimidine nucleus (111.5, 164.3, 168.2), N=CH group (161.0), OCH_3_ (54.1, 60.8, 56.1)
**Ax2**	3068	1594	1674	1272	–	723 (C–C str.), 3345 (OH str.), 593 (C–Br str., C_6_H_5_Br)	Anal calc: C, 61.65; H, 3.35; N, 8.46; Found: C, 61.69; H, 3.39; N, 8.42; *m/z*: 663	7.55-7.67 (m, 18H, Ar–H), 8.09 (s, 1H, N=CH), 7.72 (s,1H, pyrimidine)	Aromatic nucleus (113.2, 118.4, 122.6, 123.5, 124.4, 125.1, 126.6, 126.3, 128.1, 129.4, 130.2, 131.2, 133.2, 134.3, 135.3, 147.5, 154.2), pyrimidine nucleus (110.5, 163.3, 167.2), N=CH group (160.6)
**Ax3**	3075	1585	1680	1329	1243	562 (C–Br str.)	Anal calc: C, 53.54; H, 3.63; N, 7.20; Found: C, 53.56; H, 3.67; N, 7.24; *m/z*: 584	6.37-7.56 (m, 10H, Ar–H), 3.72 (s, 9H, OCH_3_), 7.89 (s, 1H, N=CH), 7.72 (s,1H, pyrimidine)	Aromatic nucleus (100.4, 112.3, 117.3, 123.0, 125.6, 126.3, 127.6, 128.1, 129.3, 130.2, 131.2, 132.2, 134.3, 139.5, 154.2), pyrimidine nucleus (110.1, 163.3, 166.2), N=CH group (161.8), OCH_3_ (55.1, 61.4, 56.1)
**Ax4**	3087	1587	1682	1327	1237	3388 (OH str.), 564 (C–Br str.)	Anal calc: C, 60.01; H, 4.26; N, 8.07; Found: C, 60.07; H, 4.30; N, 8.10; *m/z*: 521	6.92–7.77 (m, 10H, Ar–H), 3.73 (s, 9H, OCH_3_), 7.87 (s, 1H, N=CH), 7.73 (s,1H, pyrimidine)	Aromatic nucleus (100.5, 116.3, 117.3, 123.6, 123.4, 127.2, 128.1, 129.3, 130.4, 132.3, 133.2, 134.5, 139.3, 154.2, 160.2), pyrimidine nucleus (110.7, 164.1, 166.2), N=CH group (161.1), OCH_3_ (55.1, 61.4, 55.1)
**Ax5**	3069	1588	1683	1326	1238	731 (C–Cl str.), 528 (C–Br str.)	Anal calc: C, 57.96; H, 3.93; N, 7.80; Found: C, 57.92; H, 3.89; N, 7.84; *m/z*: 540	6.73–7.73 (m, 10H, Ar–H), 3.73 (s, 9H, OCH_3_), 7.88 (s, 1H, N=CH), 7.73 (s,1H, pyrimidine)	Aromatic nucleus (100.6, 112.3, 117.3, 123.4, 124.4, 127.1, 128.3, 130.4, 131.1, 132.2, 134.4, 147.5, 153.5), pyrimidine nucleus (110.5, 164.3,164.3, 167.2), N=CH group (162.0), OCH_3_ (54.1, 60.8, 56.1)
**Ax6**	2934	1589	1680	1325	1237	1345(NO_2_ str.), 850 (C-N str., NO_2_), 512 (C–Br str.)	Anal calc: C, 56.84; H, 3.85; N, 10.20; Found: C, 56.90; H, 3.89; N, 10.25; *m/z*: 550	7.56–8.18 (m, 10H, Ar–H), 3.74 (s, 9H, OCH_3_), 8.16 (s, 1H, N=CH), 7.56 (s,1H, pyrimidine)	Aromatic nucleus (100.6, 112.3, 117.3, 123.4, 124.3, 126.6, 126.3, 127.1, 128.4, 129.3, 130.2, 133.2, 134.3, 139.3, 143.5, 151.2, 154.5), pyrimidine nucleus (112.5, 165.2, 163.2), N=CH group (159.0), OCH_3_ (55.2, 61.8, 55.2)
**Ax7**	3069	1595	1675	1301	1269	3388 (OH str.)	Anal calc: C, 76.58; H, 5.00; N, 9.92; Found: C, 76.61; H, 5.06; N, 9.96; *m/z*: 566	6.55–7.63 (m, 18H, Ar–H), 3.71 (s, 3H, OCH_3_), 8.18 (s, 1H, N=CH), 7.78 (s, 1H, pyrimidine)	Aromatic nucleus (102.0, 113.3,114.4, 118.3, 122.3, 123.5, 124.4, 126.6, 126.3, 128.4, 129.3, 130.2, 133.2, 147.5, 153.2), pyrimidine nucleus (110.9, 164.3, 168.2), N=CH group (161.0), OCH_3_ (162.5, 57.1)
**Ax8**	3060	1595	1678	1270	–	2926 (C–H str. aliphatic), 1166 (C–N str. alkyl amine), 3231(OH str.)	Anal calc: C, 77.26; H, 5.80; N, 14.23; Found: C, 77.30; H, 5.84; N, 14.27; *m/z*: 592	6.78–7.70 (m, 18H, Ar–H), 2.67 (s, 12H, N(CH_3_)_2_), 8.39 (s, 1H, N=CH), 7.70 (s, 1H, pyrimidine)	Aromatic nucleus (112.3, 118.3, 122.6, 123.7, 125.4, 126.6, 126.3, 128.9, 129.3, 130.2, 133.7, 134.2, 147.5, 153.2), pyrimidine nucleus (110.5, 164.0, 167.2), N=CH group (160.6), CH_3_ (41.7, 154.9)
**Ax9**	3093	1591	1673	1363	1237	3386(OH str.), 538 (C–Br str.)	Anal calc: C, 52.11; H, 3.53; N, 7.01; Found: C, 52.15; H, 3.57; N, 7.05; *m/z*: 600	6.77–7.66 (m, 9H, Ar–H), 3.73 (s, 9H, OCH_3_), 8.19 (s, 1H, N=CH), 7.70 (s, 1H, pyrimidine)	Aromatic nucleus (102.0, 110.3, 119.3, 120.6, 123.0, 127.6, 128.0, 132.6, 134.2, 135.7, 139.0, 153.3, 160.6), pyrimidine nucleus (111.5, 164.3, 164.5, 167.2), N=CH group (159.9), OCH_3_ (55.1, 60.8, 55.1)
**Ax10**	3087	1591	1679	1328	1276	3384(OH str.), 526 (C–Br str.)	Anal calc: C, 60.01; H, 4.26; N, 8.07; Found: C, 60.05; H, 4.30; N, 8.10; *m/z*: 521	6.58–7.52 (m, 10H, Ar–H), 3.73 (s, 9H, OCH_3_), 8.20 (s, 1H, N=CH), 7.71 (s, 1H, pyrimidine)	Aromatic nucleus (105.0, 117.3, 120.5, 121.3, 123.2, 127.8, 128.4, 132.9, 132.1, 133.2, 134.8, 139.5, 153.2, 161.8), pyrimidine nucleus (111.5, 164.3, 168.2), N=CH group (161.0), OCH_3_ (55.1, 60.7, 55.1)
**Ax11**	3071	1595	1676	1360	1271	3383 (OH str.)	Anal calc: C, 76.58; H, 5.00; N, 9.92; Found: C, 76.62; H, 5.06; N, 9.96; *m/z*: 566	6.39–7.71 (m, 17H, Ar–H), 3.87 (s, 6H, OCH_3_), 8.16 (s, 1H, N=CH), 7.71 (s, 1H, pyrimidine)	Aromatic nucleus (111.3, 118.3, 121.3, 122.6, 123.8, 124.5, 126.6, 126.3, 127.7, 128.1, 129.3, 130.2, 132.6, 133.2, 134.6, 153.2, 156.9), pyrimidine nucleus (110.0, 164.3, 167.2), N=CH group (162.0), OCH_3_ (56.2)
**Ax12**	3066	1588	1685	1321	1268	712 (C–Cl str.), 628 (C–Br str.)	Anal calc: C, 57.96; H, 3.93; N, 7.80; Found: C, 57.99; H, 3.97; N, 7.84; *m/z*: 540	6.58–7.70 (m, 10H, Ar–H), 3.74 (s, 9H, OCH_3_), 7.89 (s, 1H, N=CH), 7.70 (s, 1H, pyrimidine)	Aromatic nucleus (100.6, 123.3, 126.3, 127.8, 128.1, 129.3, 130.2, 132.8, 133.9, 135.7, 138.9, 153.2), pyrimidine nucleus (110.5, 164.8, 164.3, 167.2), N=CH group (159.0), OCH_3_ (56.0, 60.6, 56.0)
**Ax13**	2959	1507	1593	1352	1239	2934 (C-H str. aliphatic), 593 (C–Br str.)	Anal calc: C, 63.40; H, 4.56; N, 7.92; Found: C, 63.45; H, 4.60; N, 7.96; *m/z*: 531	6.80–7.71 (m, 11H, Ar–H), 3.72 (s, 9H, OCH_3_), 6.80 (s, 1H, CH), 7.46 (s, 1H, N=CH), 7.71 (s, 1H, pyrimidine)	Aromatic nucleus (100.8, 123.9, 128.1, 128.5, 128.7, 132.2, 135.9, 139.5, 153.2), pyrimidine nucleus (110.5, 164.3, 164.2), N=CH group (164.0), OCH_3_ (55.1, 60.9, 55.1), CH=CH (119.0, 133.6)
**Ax14**	2970	1462	1595	1274	1241	2828 (C–H str. aliphatic), 1173 (C–N str. alkyl amine), 591 (C–Br str.)	Anal calc: C, 62.61; H, 13.88; N, 9.74; Found: C, 62.65; H, 13.84; N, 9.78; *m/z*: 576	7.51–6.74 (m, 10H, Ar–H), 3.73 (s, 9H, OCH_3_), 7.87 (s, 1H, N=CH), {3.38 (q, 2H, CH_2_), 1.14 (t, 3H, CH_3_), of N(C_2_H_5_)_2_} 7.70 (s, 1H, pyrimidine)	Aromatic nucleus (109.0, 112.3, 111.3, 123.7, 124.4, 125.8, 126.6, 126.3, 128.1, 132.2, 134.6, 148.5, 139.6, 153.2), pyrimidine nucleus (110.5, 164.3, 164.3, 167.2), N=CH group (160.0), OCH_3_ (56.1, 60.5, 56.1), N(C_2_H_5_)_2_ (12.8, 47.9)
**Ax15**	3072	1591	1694	1345	1237	528 (C–Br str.) 1416 (NO_2_ str.), 850 (C–N str., NO_2_)	Anal calc: C, 56.84; H, 3.85; N, 10.20; Found: C, 56.88; H, 3.88; N, 10.24; *m/z*: 550	6.53–8.08 (m, 10H, Ar–H), 3.73 (s, 9H, OCH_3_), 8.08 (s, 1H, N=CH), 7.91 (s, 1H, pyrimidine)	Aromatic nucleus (108.8, 123.6, 124.4, 126.3, 128.1, 129.3, 132.7, 133.2, 135.8, 139.5, 141,8, 147.5, 153.2), pyrimidine nucleus (110.5, 164.3, 167.2), N=CH group (160.0), OCH_3_ (56.0, 60.8, 56.0)
**Ax16**	3078	1462	1594	1347	1237	757 (C–Cl str.), 1410 (NO_2_ str.), 850 (C–N str., NO_2_)	Anal calc: C, 61.85; H, 4.19; N, 11.10; Found: C, 61.88; H, 4.23; N, 11.15; *m/z*: 506	6.93–8.38 (m, 10H, Ar–H), 3.73 (s, 9H, OCH_3_), 8.38 (s, 1H, N=CH), 7.70 (s, 1H, pyrimidine)	Aromatic nucleus (100.0, 124.6, 124.4, 126.6, 127.3, 128.1, 129.3, 130.2, 132.2, 133.9, 139.0, 141.5, 153.0), pyrimidine nucleus (110.8, 164.7, 164.7, 167.2), N=CH group (159.0), OCH_3_ (56.1, 60.8, 56.1)
**Ax17**	2938	1592	1666	1348	1177	3485 (C–OH str.), 1462 (NO_2_ str.), 850 (C-N str., NO_2_)	Anal calc: C, 63.39; H, 4.94; N, 10.56; Found: C, 63.43; H, 4.97; N, 10.59; *m/z*: 532	6.96–8.38 (m, 9H, Ar–H), 3.75 (s, 9H, OCH_3_), 3.31 (m, 2H, CH_2_), 1.34 (t, 3H, CH_3_), 8.38 (s, 1H, N=CH), 7.85 (s, 1H, pyrimidine)	Aromatic nucleus (100.6, 112.3, 116.3, 122.5, 123.6, 124.4, 126.3, 127.7, 128.1, 129.3, 130.2, 133.2, 139.5, 141.4, 151.6, 153.2), pyrimidine nucleus (110.5, 164.3, 14.3, 166.2), N=CH group (160.0), OCH_3_ (55.1, 6.18, 55.1), OC_2_H_5_ (14.8, 63.6)
**Ax18**	2938	1462	1550	1348	1227	1409 (NO_2_ str.), 850 (C–N str., NO_2_)	Anal calc: C, 64.79; H, 4.83; N, 11.19; Found: C, 64.72; H, 4.86; N, 11.24; *m/z*: 502	6.93–8.38 (m, 10H, Ar–H), 3.73 (s, 12H, OCH_3_), 8.38 (s, 1H, N=CH), 7.85 (s, 1H, pyrimidine)	Aromatic nucleus (100.9, 112.3, 117.3, 121.8, 124.5, 126.8, 127.3, 132.2, 139.6, 141.8, 147.5, 153.2, 157.8), pyrimidine nucleus (110.5, 164.3, 167.2), N=CH group (159.0), OCH_3_ (55.1, 60.8, 55.1, 55.0)
**Ax19**	2938	1594	1670	1348	1235	3491 (OH str.), 1276 (NO_2_ str.), 850 (C-N str., NO_2_), 583 (C–Br str.)	Anal calc: C, 55.23; H, 3.74; N, 9.91; Found: C, 55.26; H, 3.79; N, 9.95; *m/z*: 566	6.92–8.38 (m, 9H, Ar–H), 3.73 (s, 9H, OCH_3_), 8.39 (s, 1H, N=CH), 7.72 (s, 1H, pyrimidine)	Aromatic nucleus (110.3, 120.7, 124.8, 126.6, 126.3, 127.4, 132.9, 135.6, 139.6, 141.7, 147.0, 153.2), pyrimidine nucleus (110.4, 164.3, 164.3, 168.2), N=CH group (160.0), OCH_3_ (55.1, 60.0, 55.1)

### Antimicrobial screening results

**Fig. 1:**
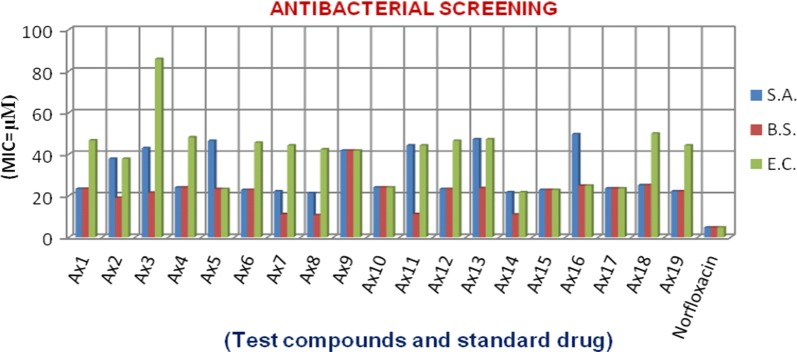
Antibacterial screening graph of synthesized compounds

**Fig. 2:**
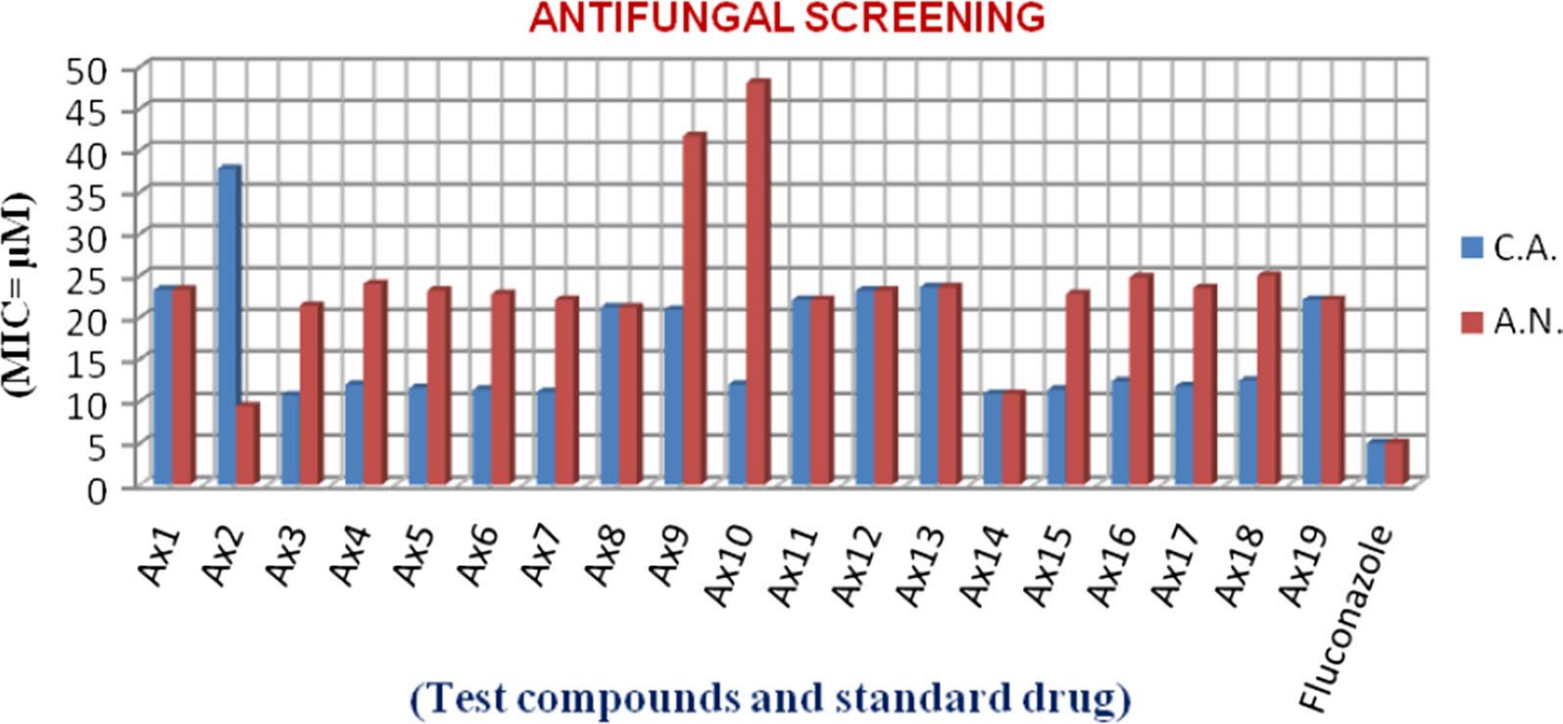
Antifungal screening graph of synthesized compounds

The pyrimidine compounds (**Ax1–Ax19**) were examined for their antimicrobial potency towards Gram −ve and Gram +ve bacteria as well as fungal species by tube dilution technique. Table 3, Figs. 1 and 2 show the antimicrobial evaluation results. The compounds showed significant antimicrobial activity than standard drugs, norfloxacin (for antibacterial study) and fluconazole (for antifungal study). In Gram negative bacteria, compound **Ax14** (MIC_*ec*_ = 21.7 µM) exhibited better antibacterial potency toward *E. coli*. In the case of Gram positive bacteria, compound **Ax8** (MIC_*sa*_ = 21.2 µM) and (MIC_*bs*_ = 10.6 µM) showed the significant potency towards *S. aureus* and *B. subtilis,* respectively. The antifungal screening results displayed that compounds, **Ax2** (MIC_*an*_ = 9.40 µM) and **Ax3** (MIC_*ca*_ = 10.7 µM) showed the significant potency towards *A. niger* and *C. albicans,* respectively. The molecules may be used as the lead compounds for the development of new antimicrobial agents.

**Table 3:** Antimicrobial activity results of synthesized heterocyclic pyrimidine derivatives

Comp.	Antimicrobial activity Minimum inhibitory concentration (MIC = µM)				
	Bacteria species (Gram+ and Gram−)			Fungal species	
	*S.A.*	*B.S.*	*E.C.*	*C.A.*	*A.N.*
**Ax1**	23.3	23.3	46.6	23.3	23.3
**Ax2**	37.8	18.9	37.8	37.8	9.40
**Ax3**	42.9	21.4	85.8	10.7	21.4
**Ax4**	24.0	24.0	48.1	12.0	24.0
**Ax5**	46.4	23.2	23.2	11.6	23.2
**Ax6**	22.8	22.8	45.5	11.4	22.8
**Ax7**	22.1	11.1	44.2	11.1	22.1
**Ax8**	21.2	10.6	42.3	21.2	21.2
**Ax9**	41.7	41.7	41.7	20.9	41.7
**Ax10**	24.0	24.0	24.0	12.0	48.1
**Ax11**	44.2	11.1	44.2	22.1	22.1
**Ax12**	23.2	23.2	46.4	23.2	23.2
**Ax13**	47.2	23.6	47.2	23.6	23.6
**Ax14**	21.7	10.9	21.7	10.9	10.9
**Ax15**	22.8	22.8	22.8	11.4	22.8
**Ax16**	49.6	24.8	24.8	12.4	24.8
**Ax17**	23.5	23.5	23.5	11.8	23.5
**Ax18**	25.0	25.0	49.9	12.5	25.0
**Ax19**	22.1	22.1	44.2	22.1	22.1
Std.	47.0^x^	47.0^x^	47.0^x^	50.0^y^	50.0^y^
DMSO	NA	NA	NA	NA	NA
Broth control	NG	NG	NG	NG	NG

Std drugs: ^x^Norfloxacin; ^y^Fluconazole; *S.A.*, *Staphylococcus aureus*; *B.S., Bacillus subtilis; E.C., Escherichia coli; C.A., Candida albicans; A.N., Aspergillus niger;* NA, no activity; NG, no growth

### Antiproliferative screening results

**Fig. 3:**
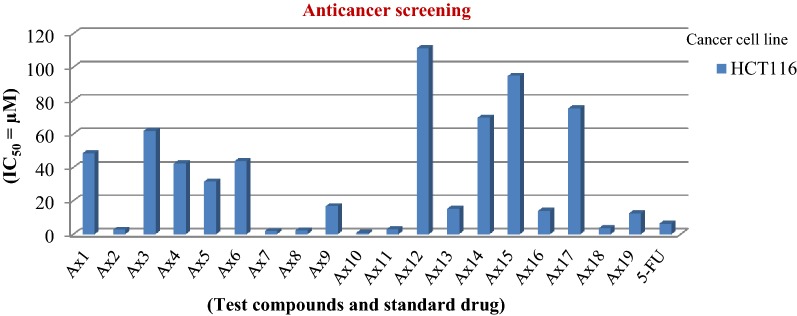
Anticancer screening graph of synthesized compounds

Table 4 and Fig. 3 show the screening results of the developed pyrimidine compounds (**Ax1–Ax19**) towards human colorectal carcinoma cell line by SRB assay [23]. The synthesized compounds exhibited good anticancer activity, with some of the findings comparable or highly potent than 5-fluorouracil (standard drug). Compounds **Ax2** (IC_50_ = 2.70 µM), **Ax7** (IC_50_ = 1.90 µM), **Ax8** (IC_50_ = 2.20 µM) and **Ax10** (IC_50_ = 0.80 µM), in particular, were the four best compounds which elicited more potent anticancer activity when compared to the reference drug (IC_50_ = 6.20 µM). They may be used as lead molecules for the development of new anticancer agent.

**Table 4:** Antiproliferative activity of synthesized pyrimidine derivatives

Anticancer activity (IC_50_ = µM)			
Comp.	Cancer cell (HCT116)	Comp.	Cancer cell (HCT116)
**Ax1**	48.4	**Ax11**	3.0
**Ax2**	2.70	**Ax12**	111.3
**Ax3**	61.7	**Ax13**	15.1
**Ax4**	42.3	**Ax14**	69.6
**Ax5**	31.5	**Ax15**	94.7
**Ax6**	43.7	**Ax16**	13.9
**Ax7**	1.90	**Ax17**	75.3
**Ax8**	2.20	**Ax18**	3.60
**Ax9**	16.7	**Ax19**	12.4
**Ax10**	0.80		
5-fluorouracil	6.20

### Molecular docking results

**Fig. 4:**
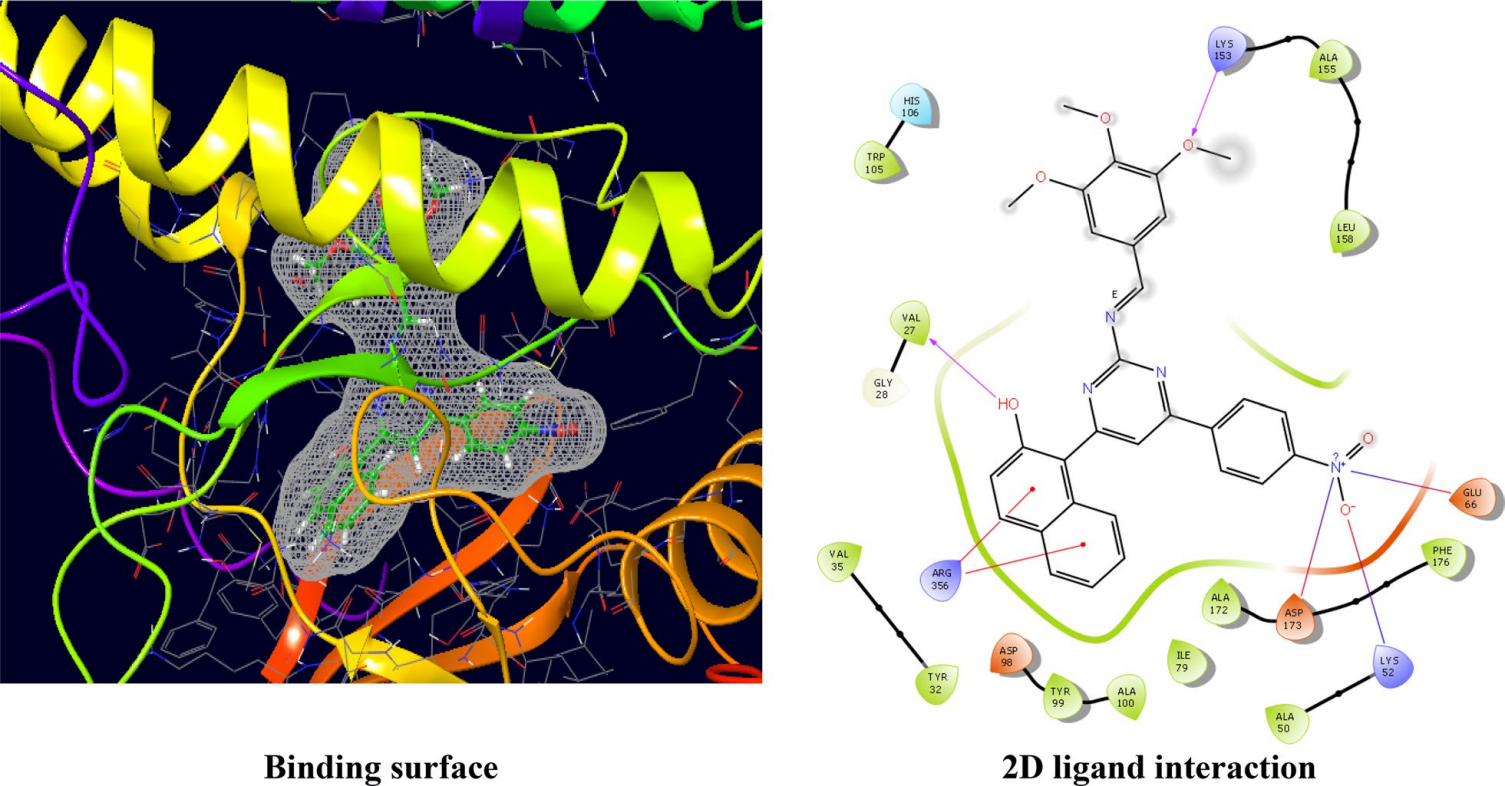
Binding surface and 2D ligand interaction diagram of compound **Ax1**

**Fig. 5:**
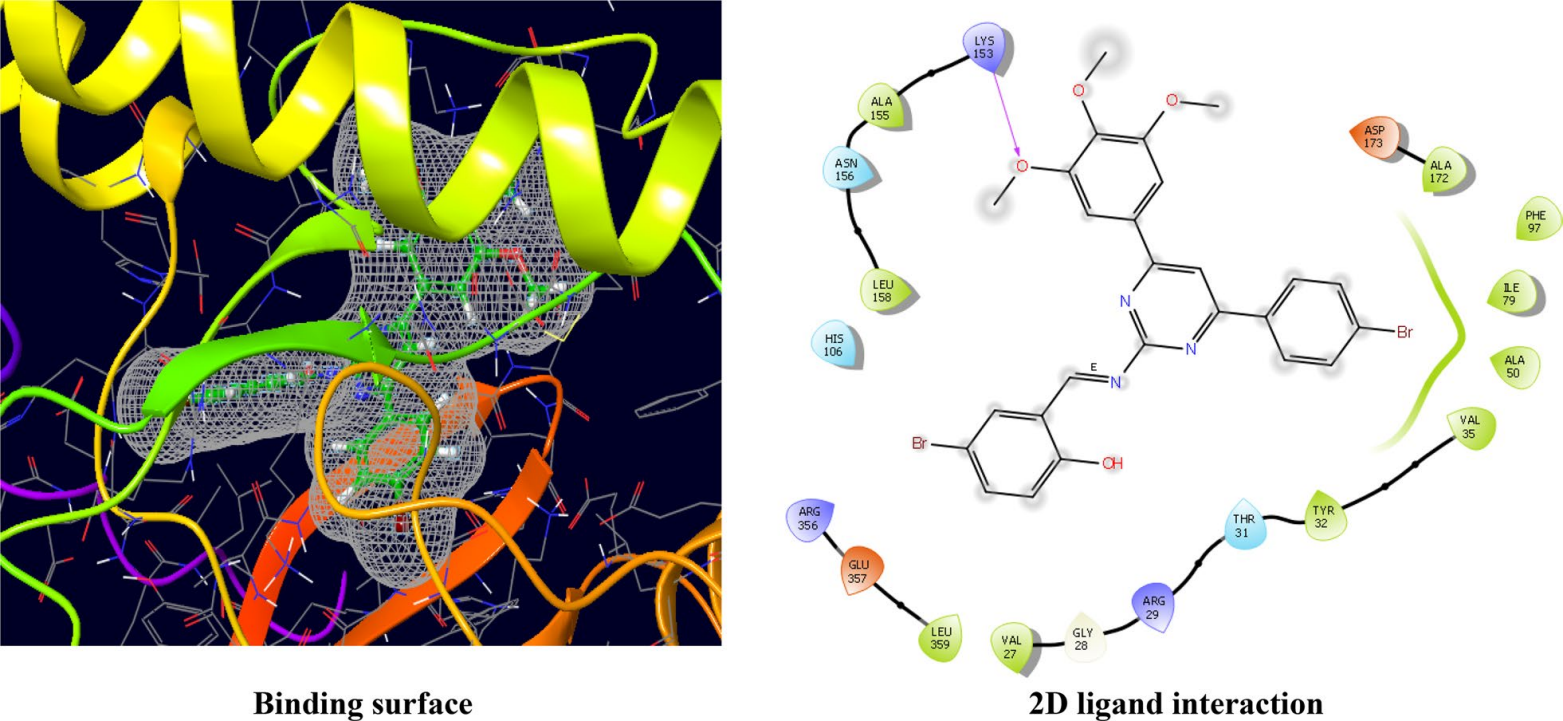
Binding surface and 2D ligand interaction diagram of compound **Ax9**

**Fig. 6:**
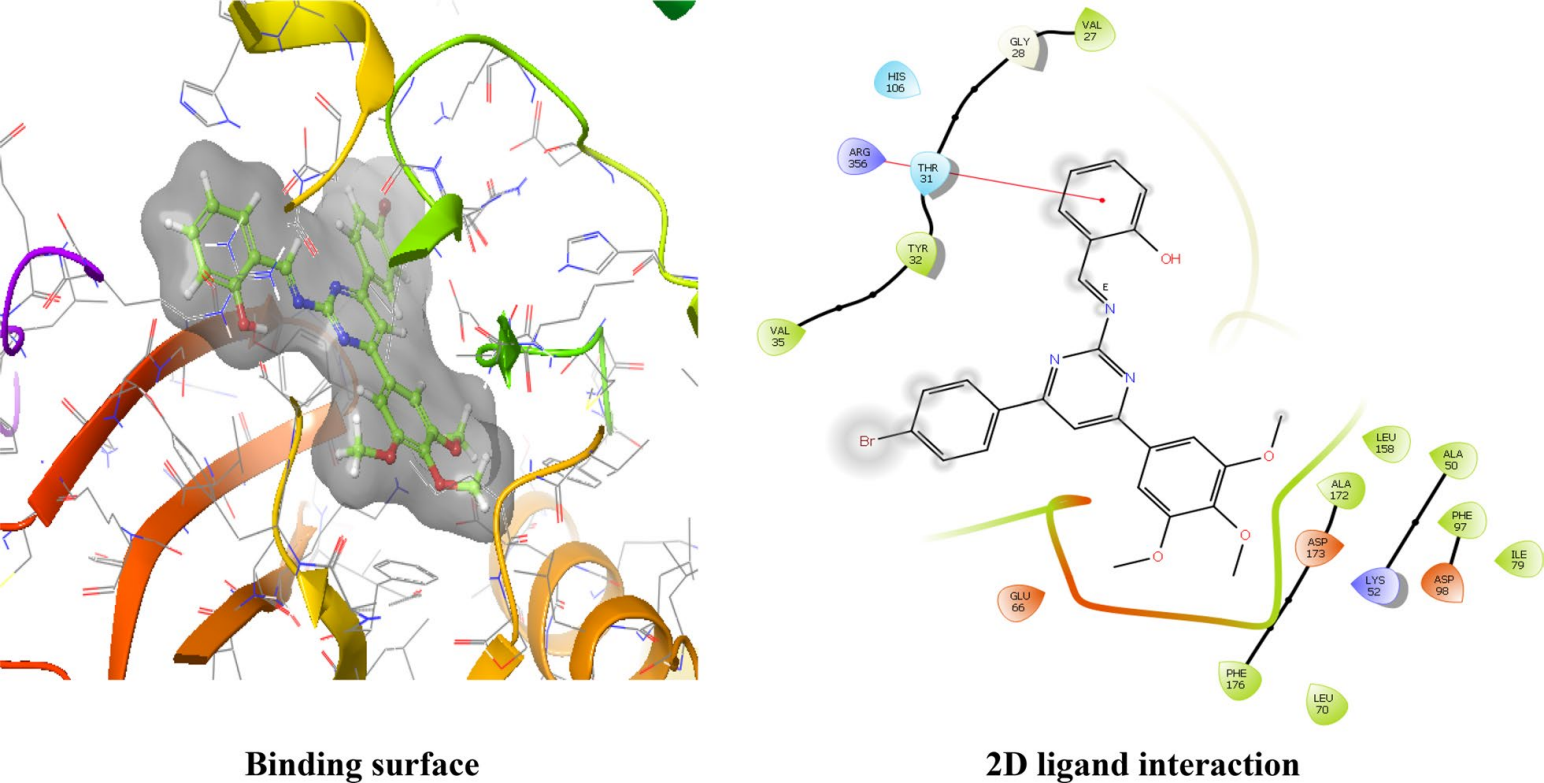
Binding surface and 2D ligand interaction diagram of compound **Ax10**

**Fig. 7:**
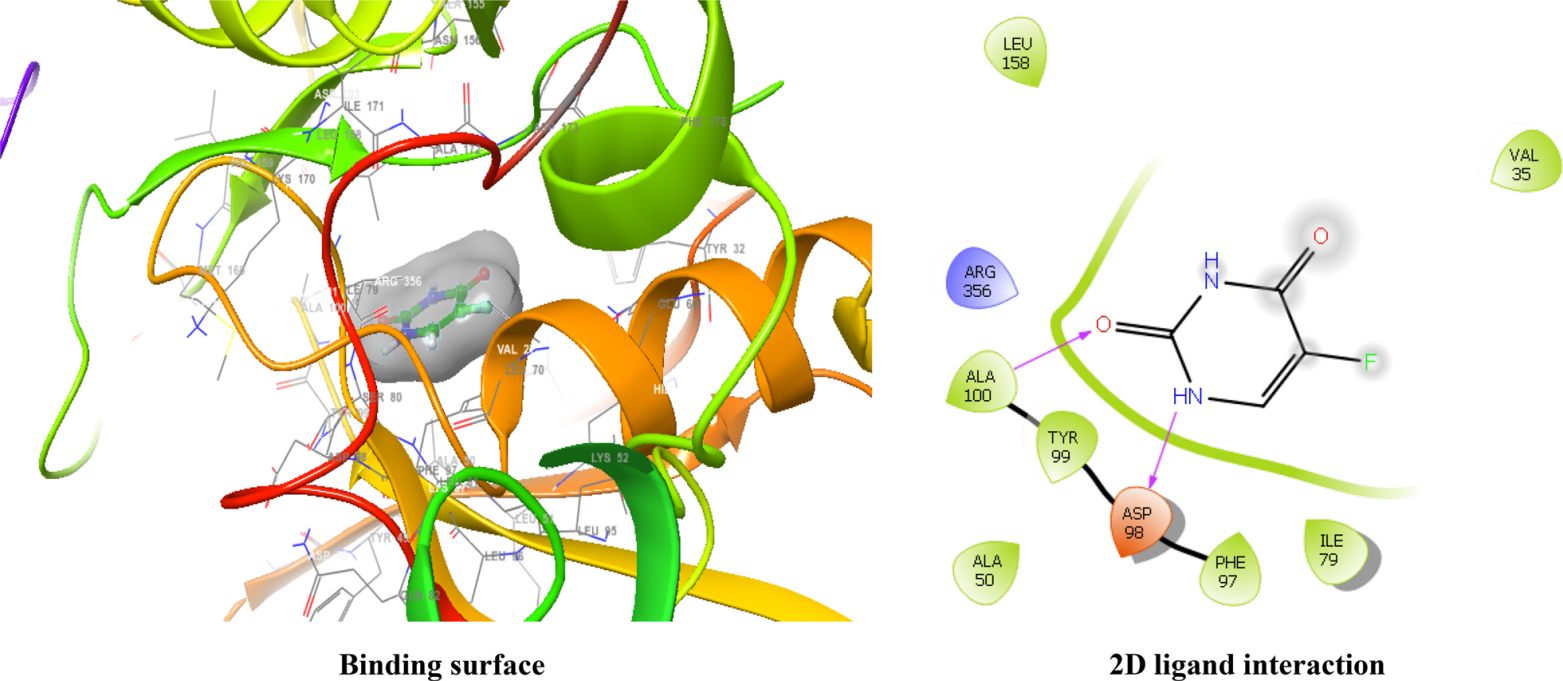
Binding surface and 2D ligand interaction diagram of 5-fluorouracil (standard drug)

The CDKs is an enzyme family that plays an significant role in the regulation of the cell cycle and thus is an especially advantageous target for the development of small inhibitory molecules [13]. The crystal structure of cyclin dependent kinase 8 (PDB Id: 5FGK) which has a good resolution of about 2.36 Å was used for docking study. The binding site of the target was generated using co-crystallized ligand (5XG) as reference (X = − 0.138, Y = − 24.891, Z = 150.623). Root-mean square deviation (RMSD) value of docked pose of native co-crystallized ligand was calculated as 0.08 Å. The synthesized pyrimidine compounds were then docked to the active site of CDK8. The docking results were analysed based on the docking score obtained from GLIDE. Among the docked compounds, compounds **Ax1**, **Ax9** and **Ax10** displayed moderate to good docked score with anticancer potency against a HCT116 cancer cell line. Ligand interaction image and binding mode of compounds **Ax1**, **Ax9** and **Ax10** in the active site of CDK8 protein having co-crystallized ligand 5XG and 5-Fu is having a different binding mode to that of active compounds (Figs. 4, 5, 6 and 7). The molecular docking results depend on the statistical evaluation function according to which the interaction energy in numerical values as docking scores [24].

Molecular docking study of the selected compounds have good to better anticancer potency toward cancer cell line were displayed moderate to better docking score within binding pocket. Binding mode of active compounds **Ax1**, **Ax9** and **Ax10** within the binding region, compound **Ax10** have moderate docked score (− 4.191) with better potency (0.80 μM) and formation of pi-cation interaction with amino acid residue Arg356; compound **Ax1** have better docked score (− 5.668) with lowest potency (48.4 μM) and formation of H-bond with amino acid residues Val27 and Lys153, pi-cation interaction with Arg356 and salt bridge with Asp173, Lys52 and Glu66 within the binding pocket and compound **Ax9** have moderate docked score (− 4.477) with moderate potency (16.7 μM) and formation of H-bond with amino acid residue Lys153 within the binding pocket and compared to 5-fluorouracil have better docked score (− 5.753) with good potency (6.20 μM) and formation of H-bond with amino acid residues Ala100 and Asp98 within binding pocket. The docking score results and interacting residues are showing in Table 5. Thus the docking analyses suggested that the pyrimidines can act as of great interest in successful chemotherapy. Cyclin dependent kinase-8 may be the target protein of pyrimidine derivatives for their antiproliferative activity.

**Table 5:** Docking results of active compounds (**Ax1**, **Ax9** and **Ax10)** and standard drug

Comp.	**Docking score**	**Glide energy (kcal/mol)**	**Glide emodel**	**XP GScore**	**Binding pocket residues**	**Interacting residues**
**Ax1**	− 5.668	− 46.167	− 68.459	− 5.668	His106, Trp105, Val27, Gly28, Val35, Tyr32, Arg356, Asp98, Tyr99, Ala100, Ile79, Ala172, Asp173, Ala50, Lys52, Phe176, Glu66, Lys153, Ala155, Leu158	H− bond interaction with Val27 and Lys153 , Pi cation interaction with Arg356, Formation of salt bridge with Asp173 and Lys52
**Ax9**	− 4.477	− 46.551	− 64.25	− 4.477	Lys153, Ala155, Asn156, Leu158, His106, Arg356, Glu357, Leu359, Val27, Gly28, Arg29, Thr31, Tyr32, Val35, Ala50, Ile79, Phe97, Asp173, Ala172	H-bond interaction with Lys153
**Ax10**	− 4.191	− 42.446	− 59.884	− 4.191	Val27, Gly28, Thr31, Tyr32, Val35, Arg356, His106, Glu66, Phe176, Asp173, Ala172, Leu158, Lys52, Ala50, Phe97, Asp98, Ile79, Leu70	Pi cation interaction with Arg356
5-fluorouracil	− 5.753	− 21.673	− 27.685	− 5.753	Leu158, Val35, Arg356, Ala100, Tyr99, Asp98, Phe97, Ile79, Ala50	H-bond interaction with Ala100 and Asp98

### SAR (structure activity relationship) study

**Fig. 8:**
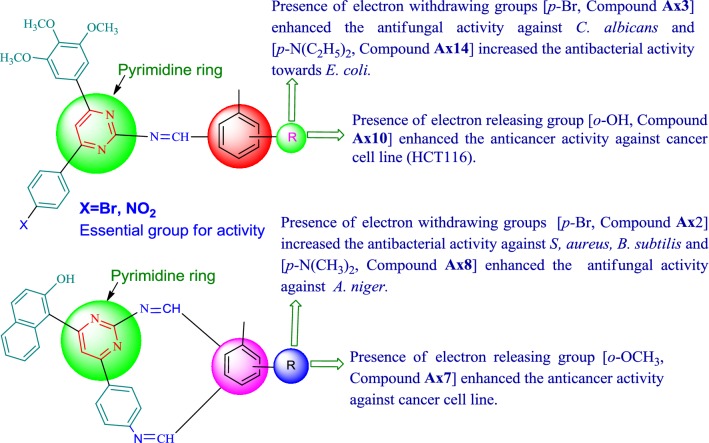
Structural activity relationship of the synthesized compounds

The following SAR can be deduced from the antimicrobial and anticancer screening results of pyrimidine analogues (Fig. 8).

### Antimicrobial activity

The presence of EWG (electron withdrawing group) (inductively)—Br at *p*-position of the substituted benzylidene aromatic nucleus of compound **Ax2** improved the antifungal activity against *A. niger* and –N(CH_3_)_2_) (an electron donating group, by mesomeric affect) at *p*-position of the benzylidene nucleus of compound **Ax8** enhanced the antibacterial activity towards *S. aureus* and *B. subtilis.*

On the other side, The presence of EWG (inductively)—Br at *p*-position of the substituted benzylidene aromatic nucleus of compound **Ax3** improved the antifungal activity toward *C. albicans* and –N(C_2_H_5_)_2_) (an electron donating group, by mesomeric affect) at *p*-position the substituted benzylidene aromatic ring of compound **Ax14** enhanced the antibacterial activity towards *E. coli.*

### Anticancer activity

The presence of EWG (inductively)—Br at *p*-position of the substituted benzylidene aromatic nucleus of compounds **Ax2** and –N(CH_3_)_2_) (an electron donating group, by mesomeric affect) at *p*-position of the substituted benzylidene aromatic ring of compound **Ax8** enhanced the anticancer activity towards a human colorectal carcinoma cell line (HCT116), however, electron releasing groups like *p*-OCH_3_ and *o*-OH on substituted benzylidene aromatic ring of compounds **Ax7** and **Ax10**, respectively showed significant role in improving the anticancer activity toward a HCT116 cell line. The SAR study is consistent the results of Kumar et al. [6, 15] and Xu et al. [25].

## Experimental

Preparatory materials were obtained from commercial sources [CDH Pvt. Ltd, HiMedia Lab. Pvt. Ltd. and Loba Chemie, Pvt Ltd. Mumbai, India] for the research work. Reaction advancement was observed by TLC (silica gel plates) using chloroform: methanol as mobile phase. Melting point was determined in open capillary tube method. Elemental analysis of the derivatives was determined by Perkin–Elmer 2400 C, H and N instrument. FTIR spectrum was recorded on Bruker 12060280 spectrometer. The Mass spectrum of the molecules was recorded on Waters Micromass Q-ToF Micro instrument. ^1^H-NMR and ^13^C-NMR were recorded at 600 MHz and 150 MHz, respectively by Bruker Avance III 600. ^1^H-NMR data are given as multiplicity and number of protons.

### Procedure for the synthesis of pyrimidine derivatives (Scheme 1, **Ax1–Ax19**)

#### (A): *Synthesis of 1*-*(2*-*(3,4,5*-*trimethoxybenzylideneamino)*-*6*-*(4*-*nitrophenyl)pyrimidin*-*4*-*yl)*-*naphthalen*-*2*-*ol* (Compound **Ax1**)

*p*-Nitroacetophenone (0.01 mol) and naphthaldehyde (0.01 mol) were added in 50 mL methanol after that 10 mL NaOH solution was added drop by drop to the reaction mixture and kept on vigorous stirring for 30 min. When the reaction mixture became turbid, it was maintained at 20–22 °C on magnetic stirrer for 4–5 h and then, the reaction mixture was neutralised by 0.1–0.2 N HCl to yield chalcone [Int-I]. The chalcone was filtered and recrystallised with methanol [26]. To the Int-I (0.01 mol), potassium hydroxide (0.01 mol) and guanidine nitrate (0.25 M) in methanol (30 mL) was added and refluxed for 5–6 h (RT). The reaction mixture was cooled and quenched with 20 mL of 0.5 M HCl solution in water to yield pyrimidine [Int-II] [27]. The Int-II (0.01 mol) was then refluxed with substituted benzaldehyde (0.01 mol) in methanol 50 mL in presence of glacial acetic acid for 2–3 h (RT). The precipitate generated by adding the reaction mixture to the ice cold water was filtered and recrystallised with methanol [28].

#### (B): *Synthesis of 1*-*(2*-*substituted benzylideneamino)*-*6*-*(4*-*substituted benzylideneamino) phenyl)pyrimidin*-*4*-*yl) naphthalen*-*2*-*ol* (Compounds **Ax2**, **Ax7**, **Ax8** and **Ax11)**

*p*-Aminoacetophenone (0.01 mol) and naphthaldehyde (0.01 mol) were added in 50 mL methanol after that 10 mL NaOH solution was added drop by drop to the reaction mixture and kept on vigorous stirring for 30 min. When the reaction mixture became turbid, it was maintained at 20–22 °C on magnetic stirrer for 4–5 h. The reaction mixture was neutralised by 0.1–0.2 N HCl to yield chalcone [Int-I]. The chalcone was filtered and recrystallised with methanol [26]. To the Int-I (0.01 mol), potassium hydroxide (0.01 mol) and guanidine nitrate (0.25 M) in methanol (30 mL) was added and refluxed for 5–6 h (RT). The reaction mixture was cooled and quenched with 20 mL of 0.5 M HCl solution in water to yield pyrimidine [Int-II] [27]. The Int-II (0.01 mol) was then refluxed with substituted benzaldehyde (0.02 mol) in methanol 50 mL in presence of glacial acetic acid for 2–3 h (RT). The precipitate generated by adding the reaction mixture to the ice cold water was filtered and recrystallised with methanol [28].

#### (C): *Synthesis of N*-*(2*-*substituted benzylidene)*-*4*-*(4*-*substituted phenyl)*-*6*-*(3,4,5*-*trimethoxy*- *phenyl)pyrimidin*-*2*-*amine* (Compounds **Ax3-Ax6**, **Ax9**, **Ax10**, **Ax12**, **Ax13**, **Ax14-Ax19**)

*p*-Substituted acetophenone (0.01 mol) and 3,4,5-trimethoxybenzaldehyde (0.01 mol) were added in 50 mL methanol after that 10 mL NaOH solution was added drop by drop to the reaction mixture and kept on vigorous stirring for 30 min. When the reaction mixture became turbid it was maintained at 20–22 °C on magnetic stirrer for 4–5 h and then, the reaction mixture was neutralised by 0.1–0.2 N HCl to yield chalcone [Int-I]. The chalcone was filtered and recrystallised with methanol [26]. To the Int-I (0.01 mol), potassium hydroxide (0.01 mol) and guanidine nitrate (0.25 M) in methanol (30 mL) was added and refluxed for 5–6 h (RT). The reaction mixture was cooled and quenched with 20 mL of 0.5 M HCl solution in water to yield pyrimidine [Int-II] [27]. The Int-II (0.01 mol) was then refluxed with substituted benzaldehyde (0.01 mol) in methanol 50 mL and added few drops of glacial acetic acid for 2–3 h (RT). The precipitate generated by adding the reaction mixture to the ice cold water was filtered and recrystallised with methanol [28].

### Biological evaluations (antimicrobial and anticancer)

The antimicrobial evaluation of developed derivatives (**Ax1-Ax19**) was carried out by tube dilution technique [29] towards Gram+ bacteria species (*S. aureus* MTCC3160; *B. subtilis* MTCC441) and Gram− ve bacterium species (*E. coli* MTCC443) and fungal species: *C. albicans* MTCC227; *A. niger* MTCC281. The stock solution of compounds and control drugs (norfloxacin and fluconazole) were prepared in DMSO to get a concentration of 100 µg/mL. Dilutions of test and reference compounds were prepared in Sabouraud dextrose broth I.P. (fungi) and double strength nutrient broth I.P. (bacteria) [30]. The test samples were incubated at 37 ± 1 °C for 48 h (*C. albicans*), at 25 ± 1 °C for 7 days (*A. niger*), 37 ± 1 °C for 24 h (bacteria) respectively and the screening results were recorded in terms of MIC. The antiproliferative potency of the developed derivatives was carried out by SRB assay [23] toward human colorectal carcinoma cancer cell line [HCT116 (ATCC CCL-247)]. Data was presented as mean IC_50_ of triplicates.

### Molecular docking

The molecular docking study was performed of the synthesized pyrimidine derivatives by GLIDE docking program of maestro *v11.5* (Schrodinger 2018-1). Among the docked compounds, compounds **Ax1**, **Ax9** and **Ax10** displayed moderate to good docked score within the binding pocket of the selected protein i.e. PDB Id: 5FGK with anticancer potency against a HCT116. The protein target for heterocyclic pyrimidine compounds was identified through the literature survey [6, 31]. Pyrimidine moiety has wide spectrum of biological potential in medicinal filed [32]. CDK8 (PDB Id: 5FGK) having native ligand 5XG (co-crystallized) with good resolution about 2.36 Å for docking study. Method: X-ray diffraction, R-value free: 0.237 [33]. The root-mean-square deviation is a measure of the average distance between the atoms of superimposed structures. RMSD value of the co-crystallized native ligand (5XG) was calculated. First, Grid is generated using ATP binding site, then docking scores are calculated (Schrodinger 2018-1, maestro *v11.5*) [34]. Ligand preparation is done using LigPrep module of maestro *v11.5.* To give the best results, the molecular structures that are docked must be good representations of the actual ligand structures as they would appear in a protein–ligand complex [35].

## Conclusion

In the present study, a series of heterocyclic pyrimidine compounds was synthesized in considerable yield and confirmed by FTIR, NMR, MS, CHN analysis. The synthesized compounds showed appreciable antimicrobial and antiproliferative activities. Structure activity relationship study indicated that compounds (**Ax2**, **Ax3**, **Ax8** and **Ax14**) having electron withdrawing and compounds (**Ax7** and **Ax10**) have electron releasing groups at substituted benzylidene aromatic nucleus exhibited significant antimicrobial and antiproliferative activities. Further, molecular docking study demonstrated that compound **Ax1** showed best docked score with lowest anticancer potency and compound **Ax10** showed the moderate docked score with better anticancer potency and compared to the 5-fluorouracil having better docked score with good anticancer potency. Cyclin dependent kinase-8 may be the target protein of heterocyclic pyrimidine compound for their antiproliferative potency. Based on the docking results it is suggested that more structural modifications are required in derivatives **Ax1** and **Ax10** to make them more potent anticancer agents and these compounds may be used as leads for the development of novel antimicrobial and anticancer agents.

## Data Availability

We have presented all our main data in the form of tables and figures.

## Abbreviations

NMR: nuclear magnetic resonance
IR: infrared
MS: mass spectrum
CHN: carbon hydrogen nitrogen
Str: starching
CADD: computer‐aided drug design
MTCC: microbial type culture collection
*E. coli*: *Escherichia coli*
*C. albicans*: *Candida albicans*
*S. aureus*: *Staphylococcus aureus*
*B. subtilis*: *Bacillus subtilis*
*A. niger*: *Aspergillus niger*
MIC: minimum inhibitory concentration
ATCC: American Type Culture Collection
HCT116: human colorectal carcinoma 116
SRB: sulforhodamine B
SAR: structure activity relationship
μM: micro mole
CDK8: cyclin dependent kinase 8
PDB: protein data bank
RMSD: root-mean-square deviation
2D: 2 dimensional
3D: 3 dimensional
RNA: ribonucleic acid
DNA: deoxyribonucleic acid
CDH: central drug house
RT: room temperature
DMSO: dimethyl sulfoxide
5-Fu: 5-fluorouracil
*O*: *ortho*
*p*: *para*
EWG: electron withdrawing group

## References

[1] Taft CA, da Silva VB, de Silva CHT (2008). Current topics in computer-aided drug design. J Pharm Sic.

[2] Kakkar S, Kumar S, Narasimhan B, Lim SM, Ramasamy K, Mani V, Shah SAA (2018). Design, synthesis and biological potential of heterocyclic benzoxazole scaffolds as promising antimicrobial and anticancer agents. Chem Cent J.

[3] Rani J, Saini M, Kumar S, Verma PK (2017). Design, synthesis and biological potentials of novel tetrahydroimidazo[1,2-a]pyrimidine derivatives. Chem Cent J.

[4] Hu Y, Fu L (2012). Targeting cancer stem cells: a new therapy to cure cancer patients. Am J Cancer Res.

[5] Kassab A, Gedawy E (2013). Synthesis and anticancer activity of novel 2-pyridyl hexahyrocyclooctathieno [2,3-*d*] pyrimidine derivatives. Eur J Med Chem.

[6] Kumar S, Lim SM, Ramasamy K, Vasudevan M, Shah SAA, Selvaraj M, Narasimhan B (2017). Synthesis, molecular docking and biological evaluation of bis-pyrimidine Schiff base derivatives. Chem Cent J.

[7] Plewczynski D, Lazniewski M, Augustyniak R, Ginalski K (2011). Can we trust docking results? Evaluation of seven commonly used programs on PDB bind database. J Comput Chem.

[8] Ece A (2019). Towards more effective acetylcholinesterase inhibitors: a comprehensive modelling study based on human acetylcholinesterase protein–drug complex. J Biomol Struct Dyn.

[9] Sherr CJ (2000). The pezcoller lecture: cancer cell cycles revisited. Cancer Res.

[10] Ece A, Sevin F (2013). The discovery of potential cyclin A/CDK2 inhibitors: a combination of 3D QSAR pharmacophore modeling, virtual screening, and molecular docking studies. Med Chem Res.

[11] Sayle KL, Bentley JF, Boyle TA, Calvert H, Cheng YZ, Curtin NJ, Endicott JA, Golding BT, Hardcastle IR, Jewsbury P, Mesguiche V, Newell DR, Noble MEM, Parsons RJ, Pratt DJ, Wang LZ, Griffin RJ (2003). Structure-based design of 2-arylamino-4-cyclohexylmethyl-5-nitroso-6-aminopyrimidine inhibitors of cyclin-dependent kinases 1 and 2. Bioorg Med Chem Lett.

[12] Ece A, Sevin F (2010). Exploring QSAR on 4-cyclohexylmethoxypyrimidines as antitumor agents for their inhibitory activity of cdk2. Lett Drug Des Discov.

[13] Peyressatre M, Prével C, Pellerano M, Morris MC (2015). Targeting cyclin-dependent kinases in human cancers: from small molecules to peptide inhibitors. Cancer.

[14] Kumar S, Narasimhan B (2018). Therapeutic potential of heterocyclic pyrimidine scaffolds. Chem Cent J.

[15] Kumar S, Lim SM, Ramasamy K, Vasudevan M, Shah SAA, Narasimhan B (2017). Bis-pyrimidine acetamides: design, synthesis and biological evaluation. Chem Cent J.

[16] Kumar S, Lim SM, Ramasamy K, Mani V, Shah SAA, Narasimhan B (2018). Design, synthesis, antimicrobial and cytotoxicity study on human colorectal carcinoma cell line of new 4,4′-(1,4-phenylene)bis(pyrimidin-2-amine) derivatives. Chem Cent J.

[17] Guo Y, Li Jing, Ma J, Yu Z, Wang H, Zhua J, Liao X, Zhao Y (2015). Synthesis and antitumor activity of *α*-aminophosphonate derivatives containing thieno[2,3-*d*] pyrimidines. Chin Chem Lett.

[18] Yejella RP, Atla SR (2011). A study of anti-inflammatory and analgesic activity of new 2,4,6-trisubstituted pyrimidines. Chem Pharm Bull.

[19] Bhalgat CM, Ali MI, Ramesh B, Ramu G (2014). Novel pyrimidine and its triazole fused derivatives: synthesis and investigation of antioxidant and anti-inflammatory activity. Arab J Chem.

[20] Ashour HM, Shaaban OG, Rizk OH, El-Ashmawy IM (2013). Synthesis and biological evaluation of thieno[2′,3′:4,5]pyrimido[1,2-*b*][1,2,4]triazines and thieno[2,3-*d*] [1,2,4]triazolo[1,5-*a*]pyrimidines as anti-inflammatory and analgesic agents. Eur J Med Chem.

[21] Meneghesso S, Vanderlinden E, Stevaert A, McGuigan C, Balzarini J, Naesens L (2012). Synthesis and biological evaluation of pyrimidine nucleoside monophosphate prodrugs targeted against influenza virus. Antivir Res.

[22] Kumar D, Khan SI, Tekwani BL, Diwan PP, Rawat S (2015). 4-Aminoquinoline–pyrimidine hybrids: synthesis, antimalarial activity, heme binding and docking studies. Eur J Med Chem.

[23] Skehan P, Storeng R, Scudiero D, Monks A, McMahon J, Vistica D, Warren JT, Bokesch H, Kenney S, Boyd MR (1990). New colorimetric cytotoxicity assay for anticancer-drug screening. J Natl Cancer Inst.

[24] Bassyouni F, El Hefnawi M, El Rashed A, Rehim MA (2017). Molecular modeling and biological activities of new potent antimicrobial, anti-inflammatory and anti-nociceptive of 5-nitro indoline-2-one derivatives. Drug Des.

[25] Xu L, Zhang Y, Dai W, Wang Y, Jiang D, Wang L, Xiao J, Yang X, Li S (2014). Design, synthesis and SAR study of novel trisubstituted pyrimidine amide derivatives as CCR26 antagonists. Molecules.

[26] Kumar N, Jain JS, Sinha R, Garg VK, Bansal SK (2009). Evaluation of some novel chalcone derivatives for antimicrobial and anti-inflammatory activity. Der Pharmacia Lettre.

[27] Asiri AM, Khan SA (2011). Synthesis and antibacterial activities of a bis-chalcone derived from thiophene and its bis-cyclized products. Molecules.

[28] Sawarkar U, Narule M, Chaudhary M (2012). Synthesis of some new 3(4-hydroxyphenyl) prop-2-en-1-one 4-phenyl substituted Schiff’s bases and their antibacterial activity. Der Pharma Chemica.

[29] Cappuccino JC, Sherman N (1999). Microbiology—a laboratory manual.

[30] Pharmacopoeia of India, vol. Ӏ (2007) Controller of Publication, Ministry of Health Department, Govt. of India, New Delhi, pp 37

[31] Kumar S, Singh J, Narasimhan B, Shah SAA, Lim SM, Ramasamy K, Mani V (2018). Reverse pharmacophore mapping and molecular docking studies for discovery of GTPase HRas as promising drug target for bis-pyrimidine derivatives. Chem Cent J.

[32] Kaur R, Kaur P, Sharma S, Singh G, Mehndiratta S, Bedi PM, Nepali K (2015). Anti-cancer pyrimidines in diverse scaffolds: a review of patent literature. Recent Pat Anti-Cancer.

[33] Amin KM, Awadalla FM, Eissa AAM, Abou- Seri AM, Hassan GS (2011). Design, synthesis and vasorelaxant evaluation of novel coumarin-pyrimidine hybrids. Bioorg Med Chem.

[34] Singh J, Kumar M, Mansuri R, Sahoo GC, Deep A (2016). Inhibitor designing, virtual screening and docking studies for methyltrans-ferase: a potential target against dengue virus. J Pharm Bioallied Sci.

[35] Driessche GVD, Fourches D (2017). Adverse drug reactions triggered by the common HLA-B*57:01 variant: a molecular docking study. J Cheminform.

